# Managing Severe Traumatic Brain Injury Across Resource Settings: Latin American Perspectives

**DOI:** 10.1007/s12028-022-01670-5

**Published:** 2023-01-12

**Authors:** Ronald Alvarado-Dyer, Sergio Aguilera, Randall M. Chesnut, Walter Videtta, Danilo Fischer, Manuel Jibaja, Daniel A. Godoy, Roxanna M. Garcia, Fernando D. Goldenberg, Christos Lazaridis

**Affiliations:** 1 Division of Neurocritical Care, Departments of Neurology, and Neurosurgery, University of Chicago Medical Center, 5841 S. Maryland Avenue, Chicago, IL 60637 USA; 2 Neurosurgery, Herminda Martin Hospital-Chillán Valparaíso University, Valparaíso, Chile; 3 Neurosurgery, University of Washington, Seattle, WA USA; 4 Intensive Care, Posadas Hospital, Buenos Aires, Argentina; 5 Intensive Care, School of Medicine, Universidad de los Andes, Santiago, Chile; 6 Intensive Care, Hospital Eugenio Espejo, School of Medicine, Universidad San Francisco de Quito, Quito, Ecuador; 7 Intensive Care, Sanatorio Pasteur, Catamarca, Argentina; 8 Neurosurgery, Feinberg School of Medicine, Northwestern University, Chicago, IL USA

**Keywords:** Traumatic brain injury, Resources, Neurosurgery, Decompressive craniectomy, Intracranial pressure

## Abstract

Severe traumatic brain injury (sTBI) is a condition of increasing epidemiologic concern worldwide. Outcomes are worse as observed in low- and middle-income countries (LMICs) versus high-income countries. Global targets are in place to address the surgical burden of disease. At the same time, most of the published literature and evidence on the clinical approach to sTBI comes from wealthy areas with an abundance of resources. The available paradigms, including the Brain Trauma Foundation guidelines, the Seattle International Severe Traumatic Brain Injury Consensus Conference, Consensus Revised Imaging and Clinical Examination, and multimodality approaches, may fit differently depending on local resources, expertise, and sociocultural factors. A first step toward addressing heterogeneity in practice is to consider comparative effectiveness approaches that can capture actual practice patterns and record short-term and long-term outcomes of interest. Decompressive craniectomy (DC) decreases intracranial pressure burden and can be lifesaving. Nevertheless, completed randomized controlled trials took place within high-income settings, leaving important questions unanswered and making extrapolations to LMICs questionable. The concept of preemptive DC specifically to address limited neuromonitoring resources may warrant further study to establish a benefit/risk profile for the procedure and its role within local protocols of care.

## Introduction

Severe traumatic brain injury (sTBI) is of global epidemiologic concern because it takes a substantial toll on health and health care costs. Consistent with other global health targets, the World Health Organization announced emergency and trauma as an essential component of United Nations universal health care coverage in 2019 before the start of the COVID-19 pandemic [1]. Global targets addressing the leading causes of neurologic disorders, including sTBI, are crucial given the growing burden of critical illness and neurosurgical disease. This is particularly relevant to low- and middle-income countries (LMICs), where sTBI is associated with double or triple the mortality risk and is anticipated to further worsen over the next decade [2–5]. Classifying countries according to income follows World Bank categories based on economic considerations [6]. Nevertheless, it is important to point out that there can be disconnect between the economy and appropriate chains of medical care. In this article, we use the terms LMICs and high-income countries (HICs) not only in terms of finances but also in this latter aspect of appropriate delivery of medical care across the spectrum of prehospital, hospital, and postdischarge care [7]. In September 2022, the fifth South American Regional Neurocritical Care Society (NCS) Conference was held in Santiago, Chile. The conference was titled “Neurointensivism in the Post-Pandemic Era,” and the purpose was to continue the mission of NCS to remain a multicontinent scientific society and, specifically, to continue engagement with practitioners in South America and the Latin American Brain Injury Consortium (LABIC). Accidental death, with sTBI as a major mechanism, is the primary cause of death among men in the 15- to 59-year age group in Latin America, with more than double the incidence as compared to HICs [8].

Here, we are reporting on a session dedicated to the management of sTBI within the context of practice in South America that focused on two topics. The first set of presentations and discussion centered on three different paradigms, namely the Brain Trauma Foundation (BTF) guidelines [9], the Consensus Revised Imaging and Clinical Examination (CREVICE) protocol [10], and multimodality neuromonitoring (MMM). The goal was to offer a brief review of the supporting literature and discuss how and where the different paradigms fit within regional and local practice patterns (Table 1 offers a synopsis). The idea of stratified resource-based protocols, in conjunction with trauma registries, is gaining increasing attention to specifically improve outcomes in resource-poor areas [11, 12]. The second topic focused on the role of decompressive craniectomy (DC) in the management of intracranial hypertension (IHT) and in the overall approach to treatment of the patient with sTBI within variable resources settings (Table 2 provides bullet points on the role of DC). In what follows, we summarize the key remarks made by participants during presentations as well as insights that emerged via the ensuing discussion with the audience, which mostly comprised intensivists and neurosurgeons practicing in various countries of South America. The overarching aim is to understand how resources shape practice and how potentially safe and effective sTBI care can be tailored in a resource-sensitive manner. In addition, we seek to continue the discussion that can lead to actionable collaborative research targets that may improve global neurotrauma care.

**Table 1:** Neurocritical care paradigms in sTBI

Paradigm	Merits	Concerns	LATAM
BTF/SIBICC	Mortality reduction (observational studies) Concise and easy to follow Long-standing and established	Generic population level thresholds Limited exploration of SBI mechanisms	Based on North American data No benefit over ICE in BEST:TRIP Limited use of ICP Rare use of PbtO_2_ Recommended where ICP is monitored
CREVICE	Studied alternative to ICP monitoring (BEST:TRIP)	Increase in brain-specific treatments Requires high clinical vigilance and frequent imaging	Developed in LATAM (internal validity) Recommended where ICP is not monitored Consider supplemental noninvasive ICP monitoring
MMM	Samples more SBI pathways (brain shock) Allows for patient-specific and dynamic adjustments	Intense and complex No robust clinical outcome data No definitive protocols	Limited by available resources Consider minimum set of feasible monitoring

BEST:TRIP, benchmark evidence from South American trials: treatment of Intracranial pressure; BTF, brain trauma foundation; CREVICE, consensus revised imaging and clinical examination; ICE, imaging and clinical examination; ICP, intracranial pressure; LATAM, Latin America; MMM, multimodality monitoring; PbtO_2_, partial brain tissue oxygen tension; SBI, secondary brain injury; SIBICC, Seattle international severe traumatic brain injury consensus conference; sTBI, severe traumatic brain injury

**Table 2:** Decompressive craniectomy for IHT in sTBI

Indications	Reasons to offer	Concerns	LATAM
IHT	Achieves ICP reduction Decreases mortality (via RESCUEicp)	Do not offer early (via DECRA) Functional outcomes can vary	DECRA and RESCUEicp performed in HICs Sociocultural considerations influence shared decision-making
Preemptive	Upfront treatment for IHT in the setting of limited neuromonitoring/ICU resources	No controlled data Potential for overuse Management of complications with limited resources Strive for early cranioplasty	Promising uncontrolled reports

DECRA, decompressive craniectomy in patients with severe traumatic brain injury; HICs, high-income countries; ICP, intracranial pressure; ICU, intensive care unit; IHT, intracranial hypertension; RESCUEicp, trial of decompressive craniectomy for traumatic intracranial hypertension; sTBI, severe traumatic brain injury

## BTF Guidelines

The BTF guidelines have been associated with a reduction in mortality [9]. Recently, a management algorithm that adopted the BTF recommendations for initial intracranial pressure (ICP) and cerebral perfusion pressure (CPP) thresholds was proposed by the Seattle International Severe Traumatic Brain Injury Consensus Conference (SIBICC) [13]. As a level IIB, the BTF recommended invasive ICP monitoring with an intervention threshold of 22 mm Hg. The BTF approach has faced several criticisms [14, 15]: (1) The methodologic approach used to derive the ICP threshold is based on a single population-level association of an ICP number with dichotomized outcomes; it does not allow differentiating between a potentially modifiable therapeutic target and a mere surrogate of severity. (2) The intervention trigger is based on unidimensional excursions over a certain number, whereas degree and duration of IHT are not considered. (3) Focusing on ICP/CPP alone ignores other crucial pathophysiologic variables that describe the relationships among cerebral blood flow, oxygen delivery and utilization, and cerebral metabolism. (4) The risk/benefit ratio of interventions to accomplish a fixed ICP goal remains underexplored. Despite these limitations, having a protocolized approach with defined intervention goals is an important merit that must be weighed against approaches that claim enhanced patient specificity at the cost of increasing implementation complexity. Contemporary data on guidelines adherence and ICP monitoring suggest that although it is the dominant approach in Europe and North America, implementation is far from universal [16–18].

The BTF guidelines are based on data from North America, and there is no evidence that they are generalizable to LMICs. In the Benchmark Evidence from South American Trials: Treatment of Intracranial Pressure (BEST:TRIP) trial, there was no outcome difference between BTF and the imaging and clinical examination (ICE) groups [19]. Furthermore, no accurate data exist on the use of ICP monitoring in South American countries. A recent Web-based survey of LABIC clinician members suggested limited use of ICP monitoring with wide variability among different countries [20]. Reasons for low use include the lack of technology secondary to financial constraints (particularly in public hospitals; usually there is no reimbursement for devices placed) and limited number of providers with expertise and ability to place invasive devices (only neurosurgeons allowed by law). More systematic data are needed to understand barriers. The most common protocols referenced were the BTF guidelines for ICP-monitored patients and CREVICE when ICP was not monitored. An important item for comparative effectiveness research in South America is understanding local practice patterns and their determinants.

### CREVICE

The lack of resources in terms of technology, personnel, and expertise in applying the BTF/SIBICC approach necessitates alternative care pathways for limited resource settings. The ICE protocol was designed for the purposes of the BEST:TRIP and guides the treatment of suspected IHT based on serial imaging and clinical examination; it is based on scheduled administration of hyperosmotic agents, with addition of other interventions (e.g., hyperventilation) within an escalating tiered approach [10, 19, 21]. In BEST:TRIP, the prespecified primary outcome, as well as all other post hoc outcomes analyses, revealed no significant differences between the ICE group and the ICP-monitored group. These results make ICE an evidence-based alternative to invasive ICP-guided approaches. Of note, the ICE group had significantly more intensive care unit (ICU) days involving brain-specific treatments; however, the median length of stay in the ICU was similar in the two groups. There were no differences in terms of serious adverse events or frequency of neurosurgical procedures or in the incidence of neurological worsening. A resource-relevant consideration is the need for frequent computed tomography (CT) scanning. The conference participants agreed that BTF guidelines should be followed in places with the technological and expertise resources for invasive ICP monitoring in eligible patients and that CREVICE is an appropriate alternative if these resources are lacking. A further question that the group thought important to explore is noninvasive means for monitoring ICP, such as transcranial Doppler ultrasound, optic nerve sheath diameter (ONSD) measurements, and quantitative pupillometry. A recent study from Nigeria explored ONSD in a rural setting and showed good performance as compared to CT for space-occupying lesions and radiographic features of IHT [22].

Recently, a group of South American intensivists and neurosurgeons generated consensus guideline recommendations on general critical care measures; no specific recommendations on neuromonitoring modalities were made [23]. An important topic for further exploration and for the design of local protocols is consideration of a minimally necessary set of neuromonitoring modalities that neurotrauma centers in LMICs could institute within context. Sustainability of such neuromonitoring modalities is a challenge that competes with other cost-effective health care solutions.

### MMM

The third paradigm discussed was MMM. The main argument for MMM is the fact that sTBI is highly heterogeneous, manifesting in multiple dynamic phenotypes with differing pathophysiology and clinical trajectories. To monitor and prevent secondary brain injury (SBI), one has to decipher mechanisms of cerebral oxidative metabolic failure [24]. Further, the impetus for considering individualization, beyond the issue of heterogeneity, is related to the idea that the relationship of ICP with clinical outcome becomes qualified when it is investigated in conjunction with other SBI parameters, such as the state of cerebrovascular pressure reactivity, quality of tissue oxygenation, and non-ICP-related metabolic and energy crisis. At the same time, there are obvious limitations to this approach, including the absence of “hard” outcome data and the fact that this is an intense and high-resource approach, with data coming out of few selected centers around the world. Brain tissue oxygenation is garnering increasing attention and is currently under study in three randomized controlled trials (RCTs), all in HICs (BONANZA [ACTRN12619001328167], BOOST-3 [NCT03754114], and OXY-TC [NCT02754063]). These trials will provide pivotal knowledge on the role that invasive brain tissue oxygenation may play in the management of sTBI. Concurrently, in the LABIC survey mentioned above, only 14.6% of responders indicated that they monitored brain oxygenation; half of them used jugular bulb oxygen saturation, and only 2% among all responders used invasive brain tissue oxygenation monitoring [20]. Along these lines, the interesting question raised is, if possible, how one can mitigate the various SBI mechanisms via less invasive and less resource-demanding approaches [25].

## DC in sTBI Across Different Clinical Environments

Understanding the role of secondary DC in sTBI has been significantly advanced by two RCTs: the Decompressive Craniectomy in Patients with Severe Traumatic Brain Injury (DECRA) trial [26] and the Trial of Decompressive Craniectomy for Traumatic Intracranial Hypertension (RESCUEicp, hereafter referred to as RESCUE) [27]. It is out of scope to delve deep into the several important differences between these two trials. Nevertheless, an important distinction should be made in how they defined “refractory” IHT as justification for DC. DECRA was a trial of early DC, whereas RESCUE is a trial of DC as a measure of last resort. The 1-year DECRA outcome data demonstrated a nonsignificant trend for worse functional outcomes in the DC group [28]. Unfavorable functional outcomes after craniectomy were 11% greater but were not significantly different from those in the control arm. Among survivors after craniectomy, there were fewer good (odds ratio 0.33; 95% confidence interval 0.12–0.91; *P* = 0.03) and more vegetative (odds ratio 5.12; 95% confidence interval 1.04–25.2; *P* = 0.04) patients. RESCUE took more than 10 years to complete across 52 hospitals in 20 countries (most patients were enrolled in the UK) [29]. At 24 months, patients in the DC group had reduced mortality (61 [33.5%] vs. 94 [54.0%]). For every 100 individuals treated surgically, 21 additional patients survived at 24 months; four were in a vegetative state, two had lower severe disability, seven had upper severe disability, five had lower moderate disability, and three had upper moderate disability. Rates of lower and upper good recovery were similar between groups. These RCTs were major undertakings requiring many years to complete across multiple countries. However, all patients included in DECRA and 91% of the patients included in RESCUE were from HICs [30]. It follows that these trials may have limited validity in LMICs, and potentially dedicated study in these settings is warranted. This is part of broader and substantial disparity between HICs and LMICs in the number of published neurosurgical trials [31].

Unresolved controversies remain despite the completion of these trials; a crucial one is whether the reduction in mortality merely translates to unacceptable long-term disability. How should these results be communicated to surrogates, and who should evaluate whether an outcome is favorable or not? Answers to these questions require engagement with surrogate decision-makers and caretakers of patients. Saliently, the sociocultural implications of surviving injury with severe disability vary considerably across cultures and countries. At some places, the concept of withdrawal of life-sustaining measures for patients unlikely to make a “meaningful” recovery is foreign both to providers and to families; at others, families are willing to allow loved ones to pass if the projected prognosis is one of poor functional recovery or makes it unlikely for social reintegration.

A different potential role for DC that may apply specifically in LMICs and for selected patients is preemptive damage control surgery [32, 33]. The rationale is to offer decompression upfront in the context of lack of resources for frequent imaging or neuromonitoring. Unpublished Latin American data from prestudy collection for BEST:TRIP revealed that almost all centers routinely admit patients with sTBI at times when ICU beds are not available, subjecting such patients to much more restricted monitoring and care (“orphan patients” or *pacientes huérfanos*). Whether DC might benefit a subpopulation of these patients is an important question. A related question, in recognition of limited access to rehabilitation services and potential high rate of loss to follow-up, is whether cranioplasty following DC might be done very early prior to discharge. There are some promising preliminary results of such an approach. Charry et al. published 4 years of experience from Neiva University Hospital, a 504‐bed level I trauma center in southern Colombia [34]. These authors reported on a cohort of 106 patients with sTBI who were operated on within 12 h from injury onset. Early decompression included a > 12-cm by 12-cm hemicraniectomy either with or without dural closure. Surgical criteria for the procedure included the following: obliteration of the basal cisterns, midline shift of > 0.5 cm, acute subdural hematoma wider than 1 cm, epidural hematomas of > 30 mL in volume, and intracerebral hemorrhage of > 50 mL in volume. Seventy-nine patients (74.6%) survived, most of them achieving a favorable neurological outcome (GOS 4–5) at 12 months after injury. Features associated with worse outcome included the following: blunt trauma, an Injury Severity Score > 16, obliterated basal cisterns, subdural hematoma as the predominant finding on CT, and nonreactive pupils on admission. Further careful study of this approach is warranted; early decompression aims to reduce the overall burden of IHT and the need for close neuromonitoring and neuroimaging. On the other side, such a practice generates concerns in relation to potential overuse, such as, performing higher number of DCs than otherwise necessary, managing complications of an early DC with limited resources, and having to strive toward early cranioplasty. An obvious issue that has to be accounted for is neurosurgical availability. A recent survey found that the average percentage of the population with access to neurosurgical services within a 2-h window in Latin America and the Caribbean is in the 60% range, with wide geographical variation [35].

## Conclusions

sTBI is a condition of increasing epidemiologic concern worldwide. Outcomes are worse as observed in LMICs vs. HICs. Global targets are in place to address the surgical burden of disease. At the same time, most of the published literature and evidence on the clinical approach to sTBI comes from wealthy areas with an abundance of resources. The available paradigms, including the BTF guidelines, SIBICC, CREVICE, and multimodality approaches, may fit differently depending on local resources, expertise, and sociocultural factors. A first step toward addressing heterogeneity in practice is to consider comparative effectiveness approaches that can capture actual practice patterns and record short-term and long-term outcomes of interest. DC decreases ICP burden and can be lifesaving. Nevertheless, completed RCTs took place within HIC settings, leaving important questions unanswered and making extrapolations to LMICs questionable. The concept of preemptive DC specifically to address limited neuromonitoring resources warrants further study to establish a benefit/risk profile for the procedure and its role within local protocols of care.
